# Relationship between fibula free flap complications and dental implant outcomes: a retrospective cohort study

**DOI:** 10.3389/froh.2025.1712296

**Published:** 2026-01-12

**Authors:** Sameh Attia, Michael Knitschke, Abanoub Riad, Mohamed Mekhemar, Kim Natalie Stolte

**Affiliations:** 1 Department of Oral and Maxillofacial Surgery, Faculty of Medicine, Justus Liebig University Giessen, Giessen, Germany; 2 Department of Periodontology, Oral Medicine and Oral Surgery, Charité—Universitätsmedizin Berlin, Berlin, Germany; 3 MKG-Dental Knitschke & Hallfeldt, Practice for Oral and Maxillofacial Surgery, Marburg, Germany; 4 Department of Public Health, Faculty of Medicine, Masaryk Centre for Global Health (MCGH), Masaryk University, Brno, Czechia; 5 Department of Public Health, Faculty of Medicine, Masaryk University, Brno, Czechia; 6 Clinic for Conservative Dentistry and Periodontology, School of Dental Medicine, Christian-Albrecht's University, Kiel, Germany

**Keywords:** fibula, fibula free flap, implants, complications, survival, function

## Abstract

**Objective:**

To clarify how fibula free flap (FFF) complications influence clinical and functional outcomes after implant-supported oral rehabilitation.

**Materials and methods:**

A single-center retrospective cohort of 29 patients (56.41 ± 12.74 years; 31% female, 69% male) who underwent segmental jaw resection, FFF reconstruction and subsequent implant placement (2002–2010) was analyzed. Demographic, oncologic, flap, implant-related and patient-centered functional variables were extracted; associations were tested with exact, rank and regression statistics (⍺ = 0.05).

**Result:**

Patients received 7.14 ± 3.06 implants each and 72% were restored with telescopic overdentures. Recipient-site wound-healing defects (WHD) occurred in 31% and were independently associated with multisegment osteotomies (*p* = 0.012) and longer operative time (*p* = 0.026); partial flap loss (PFL) was infrequent (6.9%). WHD reduced maximum inter-incisal opening (MIO) by 0.8 cm and PFL by 2.8 cm, both impairing contour ratings. Implant failure (0.59 ± 1.59) correlated with poorer speech intelligibility (*p* = 0.004) and lower aesthetic scores (*p* = 0.032). Nonetheless, 96% of patients spoke intelligibly (with or without concentration), 96% judged their dental aesthetic as good or excellent, and 75% consumed a normal diet. Ordinal regression confirmed the number of fibula segments as the sole predictor of contour (*p* = 0.001).

**Conclusions:**

FFF reconstruction permits dependable, implant-supported rehabilitation, but multisegment osteotomies and prolonged surgery heighten soft-tissue morbidity, which in turn constrains both MIO and appearance. Failed implants further degrade speech intelligibility and aesthetics. Long-term success therefore hinges on balancing the contour gains of complex osteotomies with flap vascular resilience while safeguarding implant stability.

## Introduction

Segmental mandibular defects arising from ablative surgery of benign or malign tumors, osteomyelitis, or trauma significantly impair oral function, aesthetics and quality of life, often necessitating complex reconstructive procedures (1, 2). The vascularized fibula free flap (FFF), introduced for mandible reconstruction by Hidalgo in 1989, has become widely accepted as the gold standard for mandibular reconstruction owing to its reliable vascular supply, robust cortical bone, minimal donor-site morbidity and ability to tolerate osteotomies and dental implants (3, 4).

Dental implants represent a valuable approach to oral rehabilitation in patients following cancer treatment (5–8). Although, success rates for implant in FFF-reconstructed mandibles are reported to be approximately 92%–94%, factors such as smoking and irradiated bone have been shown to significantly increase the risk of failure (9–11).

Despite favorable implant outcomes and overall flap success rates of approximately 90%, the reconstructive process remains vulnerable to complications. Wound-healing defects (WHD) at both recipient and donor site can compromise surgical success and delay or even hold up prosthetic rehabilitation (12). Donor-site complications have been characterized, with alcohol abuse and technique of donor-site closure emerging as significant risk factors for WHD following FFF harvesting (13).

Beyond surgical flap and implant outcomes, current evidence emphasizes the importance of patient-centered functional outcomes, including speech intelligibility, mastication, aesthetic perception, and overall quality of life (6, 14, 15). Here, improved health-related quality of life in patients restored with FFF and patient-specific titanium implants has been reported (16). Although not every patient undergoing mandibular reconstruction with FFF ultimately receives dental implants, satisfaction with aesthetic and functional results in implant-rehabilitated patients has been reported to be approximately 80%, highlighting dental implants as a valuable option for oral rehabilitation (15).

While recent research identifies various predictors of implantation success and complications following mandibular reconstruction, comprehensive analyses that integrate patient demographics, tumor characteristics, flap- and implant-related variables, as well as surgical and patient-centered functional outcomes, remain limited. The aim of the present study is to evaluate the relationship between fibula free flap-related complications and both clinical and functional outcomes of dental implant rehabilitation.

## Materials & methods

### Study design and setting

A single-center retrospective cohort study was conducted at the Department of Oral and Maxillofacial Surgery, University Hospital of Giessen and Marburg (UKGM), Giessen, Germany. This study was designed and reported according to the Strengthening the Reporting of Observational Studies in Epidemiology (STROBE) guidelines for cohort studies (17).

### Study period, participants, inclusion, and exclusion criteria

All patients underwent ablative surgery and FFF reconstruction with dental implant rehabilitation between January 2002 and December 2010. Inclusion criteria: the placement of at least one dental implant in the fibular graft and documentation of ≥ one follow-up visit after definitive prosthetic loading. Patients who did not receive dental implants or had mandibular reconstruction using other than FFF were not included in the study. Patients with cancer recurrence before implantation were excluded, as were those missing information on the study parameters. Standardized recall examinations were performed. As the postoperative follow-up period was not uniform across all patients, only cases with complete and well-documented follow-up data were included in the analysis, while cases with missing or incomplete data were excluded.

### Variables

Data collection encompassed the following domains: (i) sociodemographic and general anamnesis: sex, age, body mass index (BMI), tobacco and alcohol consumption, systemic comorbidities and American Society of Anesthesiologists (ASA) classification; (ii) tumor characteristics: histopathological entity, stage, defect morphology (18); (iii) FFF reconstruction characteristics: flap type, timing (immediate, delayed), number of osseous segments and osteotomies, fixation modality, operative time (min) and postoperative intensive-care-unit (ICU) stay (days); (iv) implant variables: system and number of implants (in fibula, native bone, opposite jaw), number of failed implants, prosthetic design; (v) postoperative complications: partial flap loss (PFL), recipient-site and donor-site WHD and requirement for revision surgery; (vi) patient-centered functional outcomes (patients' own self-assessment): speech intelligibility, perceived dental aesthetics, mandibular-maxillary contour, dietary consistency and maximum inter-incisal opening (MIO).

### Evaluation of patient-reported speech intelligibility, dental aesthetics, mandibular–maxillary contour, and nutritional function

Speech and dental aesthetics were assessed based on patients' self-evaluation using a structured questionnaire. The evaluation included four parameters: speech intelligibility, dental aesthetics, mandibular–maxillary contour, and nutrition.

Speech intelligibility was rated by patients as unintelligible, intelligible with concentration, or intelligible.

Dental aesthetics were self-assessed as poor, good, or excellent.

The mandibular–maxillary contour was rated as unsatisfactory, acceptable, or satisfactory.

Nutritional ability was categorized as soft diet or normal diet.

These self-assessments were collected during postoperative follow-up visits to reflect patients' subjective perception of their functional and aesthetic outcomes.

### Statistical methods

Initially, normal distribution of numerical variables, e.g., age, BMI and number of implants was tested using Shapiro–Wilk test with a significance level of *p* < 0.05. Descriptive statistics had been performed using frequencies and percentages for categorical (e.g., gender) and ordinal variables (e.g., mandibular-maxillary contour), and mean and standard deviation for numerical variables. Fisher's exact test, Mann–Whitney (U) test, Kruskal–Wallis (H) test, independent samples *t*-test, and one-way analysis of variance (ANOVA) test were used to test the hypothesized associations between independent and dependent variables. All inferential tests were conducted with a significance level of *p* < 0.05. An ordinal logistic regression model was established to examine the clinical predictors of mandibular-maxillary contour, including operative time and the number of fibula segments as independent variables. All statistical analyses were carried out using Statistical Package for the Social Sciences (SPSS) version 28 and the R-based web application Jamovi version 2.3).

### Ethics statement/confirmation of patients' permission

The Ethics committee of the faculty of medicine of Justus-Liebig University Giessen approved the study with the confirmation numbers (25/10, 5/20). Patients' consent was obtained from every included patient in the study.

## Results

### Sample characteristics

A total of twenty-nine patients were included in this study, of whom nine were females and twenty were male. The mean age at the time of surgery was 56.41 ± 12.74 years, ranging between 17.25 and 79.08 years. The mean body mass index was 26.13 ± 3.71 kg/m^2^ and was significantly higher in female than in male patients (28.17 ± 3.96 kg/m^2^ and 25.21 ± 3.29 kg/m^2^, *p* = 0.022), respectively.

**Figure 1:**
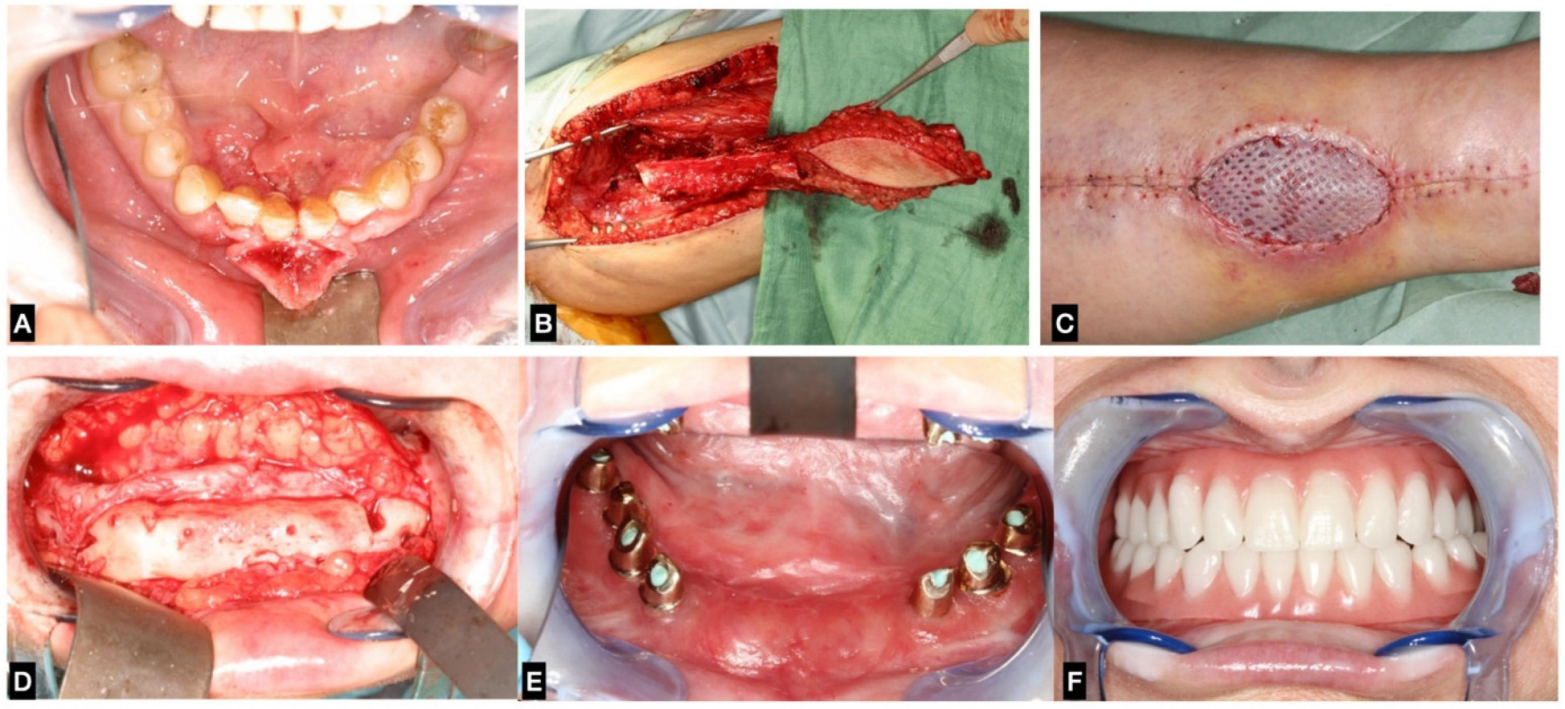
Clinical case of jaw reconstruction with FFF: **(A)** squamous cell carcinoma of the anterior mandible; **(B)** raising of the FFF; **(C)** result at donor site with well-healed skin graft being mashed; **(D)** after bone graft healing; **(E,F)** dental and oral rehabilitation achieved through dental implant placement and overdenture prosthesis.

Over one-third (34.5%) of the patients were smokers, and the most commonly reported systemic comorbidity was arterial hypertension (37.9%), followed by chronic obstructive pulmonary disease (27.6%), and coronary heart disease (10.3%) (Table 1). Figure 1 Representative clinical case included in the study, demonstrating the sequence from initial diagnosis to final dental rehabilitation.

**Table 1:** Demographic and general anamnestic characteristics of oncologic patients who underwent fibula free flap reconstruction in UKGM, January 2002–December 2010 (*n* = 29).

Variable	Outcome	Female (*n* = 9)	Male (*n* = 20)	Total (*n* = 29)	*p-value*
Age	Years: µ ± SD (Min-Max)	58.81 ± 10.75 (40.67–68.83)	55.33 ± 13.66 (17.25–79.08)	56.41 ± 12.74 (17.25–79.08)	0.253
	Months: µ ± SD (Min-Max)	705.78 ± 129.04 (488–826)	664.00 ± 163.97 (207–949)	676.97 ± 152.93 (207–949)	0.253
Body Mass Index	BMI: µ ± SD (Min-Max)	28.17 ± 3.96 (23.44–35.86)	25.21 ± 3.29 (18.61–30.86)	26.13 ± 3.71 (18.61–35.86)	**0.022**
Tobacco Smoking	Yes (*n*)	3 (33.3%)	7 (35.0%)	10 (34.5%)	1.000
Alcohol Drinking	Yes (*n*)	0 (0%)	2 (10%)	2 (6.9%)	1.000
Arterial Hypertension	Yes (*n*)	2 (22.2%)	9 (45.0%)	11 (37.9%)	0.412
Coronary Heart Disease	Yes (*n*)	0 (0%)	3 (15.0%)	3 (10.3%)	0.532
COPD	Yes (*n*)	1 (11.1%)	7 (35.0%)	8 (27.6%)	0.371
Diabetes Mellitus	Yes (*n*)	0 (0%)	2 (10.0%)	2 (6.9%)	1.000
Thyroid Gland Disorder	Yes (*n*)	1 (11.1%)	1 (5.0%)	2 (6.9%)	0.532

Mann–Whitney (U), independent samples *t*-test, and Fisher's exact test were used with a significance level of *p* < 0.05.

Bold values indicate statistically significant results.

### Tumor-related characteristics

The predominant diagnosis was squamous-cell carcinoma (72.4%), followed by adenosquamous carcinoma (6.9%), ameloblastoma (6.9%), and osteomyelitis (6.9%). Tumor staging was assessed according to the AJCC TNM Classification, 7th edition, and revealed comparable results between female and male patients, with a mean T stage of 2.87 ± 1.36 and 3.07 ± 1.00, respectively, and a mean N stage of 0.75 ± 0.89 and 0.36 ± 0.75, respectively. No statistically significant sex-related differences were observed for either T or N stage. The mean bone defect length was 7.86 ± 1.94 cm, ranging between 4.5 and 12 cm, whereas the mean mucosal defect length and width were 6.64 ± 2.50 cm and 2.92 ± 1.08 cm, respectively. Evaluation of general health status using the American Society of Anesthesiologists (ASA) classification revealed comparable scores for female and male patients (2.33 ± 0.71 and 2.05 ± 0.74, respectively; Table 2).

**Table 2:** Tumor-related characteristics of oncologic patients who underwent fibula free flap reconstruction in UKGM, January 2002–December 2010 (*n* = 29).

Variable	Outcome	Female (*n* = 9)	Male (*n* = 20)	Total (*n* = 29)	*p-value*
Tumor Type	Squamous Cell Carcinoma	6 (66.7%)	15 (75.0%)	21 (72.4%)	0.527
	Adenosquamous Carcinoma	1 (11.1%)	1 (5.0%)	2 (6.9%)	
	Mucoepidermoid Carcinoma	1 (11.1%)	0 (0%)	1 (3.4%)	
	Ameloblastoma	0 (0%)	2 (10.0%)	2 (6.9%)	
	Osteomyelitis	1 (11.1%)	1 (5.0%)	2 (6.9%)	
	Keratocystic Odontogenic Tumor	0 (0%)	1 (5.0%)	1 (3.4%)	
Stage	T: µ ± SD (Min-Max)	2.87 ± 1.36 (1–4)	3.07 ± 1.00 (2–4)	3.00 ± 1.11 (1–4)	0.764
	N: µ ± SD (Min-Max)	0.75 ± 0.89 (0–2)	0.36 ± 0.75 (0–2)	0.50 ± 0.80 (0–2)	0.440
Bone Defect	Length in cm: µ ± SD (Min-Max)	7.50 ± 1.90 (5–11)	8.03 ± 1.98 (4.5–12)	7.86 ± 1.94 (4.5–12)	0.255
Mucosa Defect	Length in cm: µ ± SD (Min-Max)	5.57 ± 2.88 (3–10)	7.06 ± 2.29 (3–11)	6.64 ± 2.50 (3–11)	0.094
	Width in cm: µ ± SD (Min-Max)	3.00 ± 1.00 (2–4)	2.89 ± 1.13 (2–6)	2.92 ± 1.08 (2–6)	0.701
Overall Health	ASA: µ ± SD (Min-Max)	2.33 ± 0.71 (1–3)	2.05 ± 0.76 (1–3)	2.14 ± 0.74 (1–3)	0.390

Mann–Whitney (U), independent samples *t*-test, and Fisher's exact test were used with a significance level of *p* < 0.05.

### Flap-related characteristics

Approximately three-quarters of the patients (75.9%) received osteomyocutaneous fibula flaps, whereas the remaining 24.1% were reconstructed using flaps without cutis. Twenty-one patients (72.4%) underwent immediate reconstruction. The most common fixation method was miniplates (MP) (82.8%), followed by reconstruction plates (RP) (10.3%) and wires (6.9%). Fewer than one quarter (24.1%) of the patients received three-segment fibula flaps, whereas 34.5% received two-segment and 41.4% single-segment flaps. The average number of segments was 1.83 ± 0.81, with no significant difference between female and male patients (2 ± 0.87 and 1.75 ± 0.79, *p* = 0.501), respectively.

The average operative time was 518.59 ± 111.41 min ranging between 313 and 764 min, with no statically significant difference between male and female patients (547 ± 115.40, 510.30 ± 111.59, *p* = 0.280), respectively. The average duration of stay at intensive care (ICU) was 1.79 ± 1.80 days (Table 3).

**Table 3:** Fibula free flap (FFF)-related characteristics of oncologic patients who underwent fibula free flap reconstruction in UKGM, January 2002–December 2010 (*n* = 29).

Variable	Outcome	Female (*n* = 9)	Male (*n* = 20)	Total (*n* = 29)	*p-value*
Flap Type	Osteomyocutaneous	6 (66.7%)	16 (80.0%)	22 (75.9%)	0.642
	Without Cutis	3 (33.3%)	4 (20.0%)	7 (24.1%)	
Reconstruction	Immediate	6 (66.7%)	15 (75.0%)	21 (72.4%)	0.675
	Delayed	3 (33.3%)	5 (25.0%)	8 (27.6%)	
Fixation Method	Miniplates	6 (66.7%)	18 (90.0%)	24 (82.8%)	0.191
	Reconstruction plates	2 (22.2%)	1 (5.0%)	3 (10.3%)	
	Wire	1 (11.1%)	1 (5.0%)	2 (6.9%)	
Fibula Segments	One Segment	3 (33.3%)	9 (45.0%)	12 (41.4%)	0.779
	Two Segments	3 (33.3%)	7 (35.0%)	10 (34.5%)	
	Three Segments	3 (33.3%)	4 (20.0%)	7 (24.1%)	
	*N*: µ ± SD (Min-Max)	2.00 ± 0.87 (1–3)	1.75 ± 0.79 (1–3)	1.83 ± 0.81 (1–3)	0.501
Fibula Osteotomies	Zero Osteotomies	3 (33.3%)	8 (40.0%)	11 (37.9%)	0.183
	One Osteotomy	6 (66.7%)	7 (35.0%)	13 (44.8%)	
	Two Osteotomies	0 (0%)	5 (25.0%)	5 (17.2%)	
	*N*: µ ± SD (Min-Max)	0.67 ± 0.50 (0–1)	0.85 ± 0.81 (0–2)	0.79 ± 0.73 (0–2)	0.694
Operative Time	Minutes: µ ± SD (Min-Max)	547.00 ± 115.40 (355–735)	510.30 ± 111.59 (313–764)	518.59 ± 111.41 (313–764)	0.280
ICU Duration	Days: µ ± SD (Min-Max)	2.11 ± 2.98 (1–10)	1.65 ± 0.99 (1–5)	1.79 ± 1.80 (1–10)	0.444

Mann–Whitney (U), independent samples *t*-test, and Fisher's exact test were used with a significance level of *p* < 0.05.

### Implant-related characteristics

The most utilized implant system was Xive (DENTSPY SIRONA Inc., Charlotte, North Carolina, USA) (51.7%), followed by BEGO (BEGO GmbH & Co. KG, Bremen, Germany) (37.9%), and Straumann (Straumann Group, Basel, Switzerland) (10.3%). Twenty-one patients (72.4%) were rehabilitated with telescopic dentures and eight (27.6%) received fixed bridges.

The average total number of inserted implants was 7.14 ± 3.06 (range 3–14); female patients received fewer implants than male patients (6.44 ± 2.35, 7.45 ± 3.33), respectively. On average, 4.03 ± 1.99 implants were inserted into the fibular graft, 1.72 ± 1.89 into native bone, and 1.38 ± 2.78 into the opposing jaw. The majority of implants (6.03 ± 4.02) were in function, whereas 0.14 ± 0.35 remained dormant (sleeping implants). Female patients not only had fewer implants in function (4.56 vs. 6.70), but also more failed implants (1.67 vs. 0.10) than their male counterparts (Table 4).

**Table 4:** Implant-related characteristics of oncologic patients who underwent fibula free flap reconstruction in UKGM, January 2002—December 2010 (*n* = 29).

Variable	Outcome	Female (*n* = 9)	Male (*n* = 20)	Total (*n* = 29)	*p-value*
Implant System	BEGO®	5 (55.6%)	6 (30.0%)	11 (37.9%)	0.385
	Xive®	4 (44.4%)	11 (55.0%)	15 (51.7%)	
	Straumann®	0 (0%)	3 (15.0%)	3 (10.3%)	
Prosthetic Design	Fixed Bridge	2 (22.2%)	6 (30.0%)	8 (27.6%)	1.000
	Telescopic Denture	7 (77.8%)	14 (70%)	21 (72.4%)	
Number of Implants	Implants in Fibula: µ ± SD (Min-Max)	3.67 ± 1.80 (0–6)	4.20 ± 2.09 (0–8)	4.03 ± 1.99 (0–8)	0.257
	Implants in Native Jaw: µ ± SD (Min-Max)	2.11 ± 2.09 (0–7)	1.55 ± 1.82 (0–6)	1.72 ± 1.89 (0–7)	0.417
	Implants in Oppo. Jaw: µ ± SD (Min-Max)	0.67 ± 2.00 (0–6)	1.70 ± 3.05 (0–8)	1.38 ± 2.78 (0–8)	0.532
	Sleeping Implants: µ ± SD (Min-Max)	0.22 ± 0.44 (0–1)	0.10 ± 0.31 (0–1)	0.14 ± 0.35 (0–1)	0.627
	Implants in Function: µ ± SD (Min-Max)	4.56 ± 3.68 (0–12)	6.70 ± 4.08 (0–14)	6.03 ± 4.02 (0–14)	0.095
	Failed Implants: µ ± SD (Min-Max)	1.67 ± 2.55 (0–6)	0.10 ± 0.45 (0–2)	0.59 ± 1.59 (0–6)	0.095
	Total Implants: µ ± SD (Min-Max)	6.44 ± 2.35 (4–12)	7.45 ± 3.33 (3–14)	7.14 ± 3.06 (3–14)	0.594

Mann–Whitney (U), independent samples *t*-test, and fisher's exact test were used with a significance level of *p* < 0.05.

### Flap-related complications

Two patients (6.9%) experienced PFL. A total of nine patients (31%) presented with WHD at the recipient site, and nine (31%) likewise exhibited defects at the donor site. Five patients (17.2%) required revision surgeries. No statistically significant differences were observed between male and female patients in PFL, WHD and revision surgery (*p* = 0.532, 0.396, and *1.000, respectively*, Table 5).

**Table 5:** Fibula free flap (FFF)-related complications of oncologic patients who underwent fibula free flap reconstruction in UKGM, January 2002–December 2010 (*n* = 29).

Variable	Outcome	Female (*n* = 9)	Male (*n* = 20)	Total (*n* = 29)	*p-value*
Partial Flap Loss	Yes (*n*)	1 (11.1%)	1 (5.0%)	2 (6.9%)	0.532
Wound Healing Defect	Recipient Site: Yes (*n*)	4 (44.4%)	5 (25.0%)	9 (31.0%)	0.396
	Donor Site: Yes (*n*)	4 (44.4%)	5 (25.0%)	9 (31.0%)	0.396
Revision Surgery	Yes (*n*)	1 (11.1%)	4 (20.0%)	5 (17.2%)	1.000

Fisher's exact test was used with a significance level of *p* < 0.05.

On evaluating the preemptive risk factors of WHD at recipient site, age, BMI, tobacco smoking, alcohol consumption, systemic comorbidities, tumor type, tumor stage, and bone and mucosal defect dimensions had no significant associations with WHD at recipient sites. The mean ASA class was slightly lower in patients with WHD than in those without (1.89 vs. 2.25). Delayed reconstruction (44.4% vs. 20%) and flaps without cutis (44.4% vs. 15%) were associated with a higher incidence of WHD. A greater number of fibula segments and a longer operative time were also significantly correlated with WHD (*p* = 0.012 and 0.028, respectively). The number of implants was not significantly associated with WHD at recipient site (Table 6).

**Table 6:** Risk factors of fibula free flap (FFF)-related complications of oncologic patients who underwent fibula free flap reconstruction in UKGM, January 2002–December 2010 (*n* = 29).

Parameters	Variable	Outcome	Wound Healing Defect: Recipient Site		*p-value*	Wound Healing Defect: Donor Site		*p-value*
			Yes (*n* = 9)	No (*n* = 20)		Yes (*n* = 9)	No (*n* = 20)	
Demographic Characteristics	Age	Years: (µ ± SD)	54.84 ± 8.68	57.12 ± 14.35	0.332	63.19 ± 8.40	53.36 ± 13.34	**0** **.** **026**
		Months: (µ ± SD)	658.11 ± 104.19	685.45 ± 172.20	0.332	758.33 ± 100.80	640.35 ± 160.13	**0** **.** **026**
	Body Mass Index	BMI: (µ ± SD)	26.34 ± 3.75	26.03 ± 3.79	0.419	26.02 ± 3.72	26.17 ± 3.80	0.460
	Tobacco Smoking	Yes (*n*)	3 (33.3%)	7 (35%)	1.000	3 (33.3%)	7 (35%)	1.000
	Alcohol Drinking	Yes (*n*)	1 (11.1%)	1 (5%)	0.532	0 (0%)	2 (10%)	1.000
	Arterial Hypertension	Yes (*n*)	2 (22.2%)	9 (45%)	0.412	3 (33.3%)	8 (40%)	1.000
	CHD	Yes (*n*)	1 (11.1%)	2 (10%)	1.000	0 (0%)	3 (15%)	0.532
	COPD	Yes (*n*)	1 (11.1%)	7 (35%)	0.371	3 (33.3%)	5 (25%)	0.675
	Diabetes Mellitus	Yes (*n*)	0 (0%)	2 (10%)	1.000	0 (0%)	2 (10%)	1.000
	Thyroid G. Disorder	Yes (*n*)	2 (22.2%)	0 (0%)	0.089	1 (11.1%)	1 (5%)	0.532
Tumor-related Characteristics	Tumor Type	Squamous Cell Carcinoma	6 (66.7%)	15 (75%)	0.277	8 (88.9%)	13 (65%)	0.397
		Adenosquamous Carcinoma	0 (0%)	2 (10%)		0 (0%)	2 (10%)	
		Mucoepidermoid Carcinoma	1 (11.1%)	0 (0%)		1 (11.1%)	0 (0%)	
		Ameloblastoma	0 (0%)	2 (10%)		0 (0%)	2 (10%)	
		Osteomyelitis	1 (11.1%)	1 (5%)		0 (0%)	2 (10%)	
		Keratocystic O. Tumor	1 (11.1%)	0 (0%)		0 (0%)	1 (5%)	
	Stage	T: (µ ± SD)	3.14 ± 1.22	2.93 ± 1.10	0.731	3.11 ± 1.17	2.92 ± 1.12	0.744
		N: (µ ± SD)	0.43 ± 0.79	0.53 ± 0.83	0.837	0.33 ± 0.71	0.62 ± 0.87	0.512
	Bone Defect	Length in cm: (µ ± SD)	7.33 ± 1.22	8.10 ± 2.17	0.166	7.56 ± 1.79	8.00 ± 2.03	0.288
	Mucosa Defect	Length in cm: (µ ± SD)	6.00 ± 3.06	6.89 ± 2.30	0.218	7.44 ± 2.60	6.19 ± 2.40	0.117
		Width in cm: (µ ± SD)	2.57 ± 0.79	3.06 ± 1.16	0.423	3.00 ± 1.00	2.88 ± 1.15	0.677
	Overall Health	ASA: (µ ± SD)	1.89 ± 0.60	2.25 ± 0.79	0.234	2.44 ± 0.73	2.00 ± 0.73	0.167
Fibula Free Flap (FFF)-related Characteristics	Flap Type	Osteomyocutaneous	5 (55.6%)	17 (85%)	0.158	9 (100%)	13 (65%)	0.066
		Without Cutis	4 (44.4%)	3 (15%)		0 (0%)	7 (35%)	
	Reconstruction	Immediate	5 (55.6%)	16 (80%)	0.209	8 (88.9%)	13 (65%)	0.371
		Delayed	4 (44.4%)	4 (20%)		1 (11.1%)	7 (35%)	
	Fixation Method	MP	7 (77.8%)	17 (85%)	0.295	7 (77.8%)	17 (85%)	0.780
		RP	2 (22.2%)	1 (5%)		1 (11.1%)	2 (10%)	
		Wire	0 (0%)	2 (10%)		1 (11.1%)	1 (5%)	
	Fibula Segments	One Segment	5 (55.6%)	7 (35%)	**0** **.** **012**	5 (55.6%)	7 (35%)	0.461
		Two Segments	0 (0%)	10 (50%)		3 (33.3%)	7 (35%)	
		Three Segments	4 (44.4%)	3 (15%)		1 (11.1%)	6 (30%)	
		*N*: (µ ± SD)	1.89 ± 1.05	1.80 ± 0.70	0.945	1.56 ± 0.73	1.95 ± 0.83	0.274
	Fibula Osteotomies	Zero Osteotomies	5 (55.6%)	6 (30%)	0.122	4 (44.4%)	7 (35%)	1.000
		One Osteotomy	3 (33.3%)	10 (50%)		4 (44.4%)	9 (45%)	
		Two Osteotomies	1 (11.1%)	4 (20%)		1 (11.1%)	4 (20%)	
		*N*: (µ ± SD)	0.56 ± 0.73	0.90 ± 0.72	0.274	0.67 ± 0.71	0.85 ± 0.75	0.594
	Operative Time	Minutes: (µ ± SD)	577.33 ± 123.63	492.15 ± 97.39	**0** **.** **028**	520 ± 102.97	517.95 ± 117.58	0.482
	ICU Duration	Days: (µ ± SD)	2.22 ± 2.95	1.60 ± 1.00	0.871	2.67 ± 3.04	1.40 ± 0.60	0.501
Implant-related Characteristics	Implant System	BEGO®	2 (22.2%)	9 (45%)	0.618	5 (55.6%)	6 (30%)	0.385
		Xive®	6 (66.7%)	9 (45%)		3 (33.3%)	12 (60%)	
		Straumann®	1 (11.1%)	2 (10%)		1 (11.1%)	2 (10%)	
	Prosthetic Design	Fixed Bridge	3 (33.3%)	5 (25%)	0.675	3 (33.3%)	5 (25%)	0.675
		Telescopic Denture	6 (66.7%)	15 (75%)		6 (66.7%)	15 (75%)	
	Number of Implants	In Fibula: (µ ± SD)	3.78 ± 1.99	4.15 ± 2.03	0.325	3.44 ± 2.01	4.30 ± 1.98	0.146
		In Native Jaw: (µ ± SD)	1.89 ± 2.26	1.65 ± 1.76	0.379	2.22 ± 2.33	1.50 ± 1.67	0.175
		In Oppo. Jaw: (µ ± SD)	3.00 ± 3.61	0.65 ± 2.01	0.140	0.78 ± 2.33	1.65 ± 2.96	0.594
		Sleeping Implants: (µ ± SD)	N/A	0.20 ± 0.41	0.417	0.33 ± 0.50	0.05 ± 0.22	0.234
		In Function: (µ ± SD)	7.89 ± 5.01	5.20 ± 3.30	0.084	5.67 ± 3.16	6.20 ± 4.42	0.374
		Failed Implants: (µ ± SD)	0.78 ± 1.99	0.50 ± 1.43	0.799	0.44 ± 0.88	0.65 ± 1.84	0.799
		Total Implants: (µ ± SD)	8.67 ± 4.06	6.45 ± 2.28	0.078	6.44 ± 2.79	7.45 ± 3.19	0.211
	Implant Survival	Failure	2 (22.2%)	3 (15%)	0.633	2 (22.2%)	3 (15%)	0.633
		Survival	7 (77.8%)	17 (85%)		7 (77.8%)	17 (85%)	

Parameters	Variable	Outcome	Partial Flap Loss		*p-value*	Revision Surgery		*p-value*
			Yes (*n* = 2)	No (*n* = 27)		Yes (*n* = 5)	No (*n* = 24)	
Demographic and General Anamnesis	Age	Years: (µ ± SD)	63.08 ± 0.47	55.92 ± 13.09	0.227	62.40 ± 4.33	55.17 ± 13.60	0.128
		Months: (µ ± SD)	757 ± 5.66	671.04 ± 157.03	0.227	748.80 ± 51.91	662 ± 163.25	0.128
	Body Mass Index	BMI: (µ ± SD)	29.25 ± 2.28	25.90 ± 3.72	0.112	23.66 ± 4.20	26.64 ± 3.48	0.052
	Tobacco Smoking	Yes (*n*)	1 (50%)	9 (33.3%)	1.000	3 (60%)	7 (29.2%)	0.306
	Alcohol Drinking	Yes (*n*)	0 (0%)	2 (7.4%)	1.000	0 (0%)	2 (8.3%)	1.000
	Arterial Hypertension	Yes (*n*)	1 (50%)	10 (37%)	1.000	2 (40%)	9 (37.5%)	1.000
	CHD	Yes (*n*)	0 (0%)	3 (11.1%)	1.000	0 (0%)	3 (12.5%)	1.000
	COPD	Yes (*n*)	1 (50%)	7 (25.9%)	0.483	2 (40%)	6 (25%)	0.597
	Diabetes Mellitus	Yes (*n*)	0 (0%)	2 (7.4%)	1.000	0 (0%)	2 (8.3%)	1.000
	Thyroid G. Disorder	Yes (*n*)	0 (0%)	2 (7.4%)	1.000	1 (20%)	1 (4.2%)	0.320
Tumor-related Characteristics	Tumor Type	Squamous Cell Carcinoma	2 (100%)	19 (70.4%)	1.000	4 (80%)	17 (70.8%)	0.526
		Adenosquamous Carcinoma	0 (0%)	2 (7.4%)		0 (0%)	2 (8.3%)	
		Mucoepidermoid Carcinoma	0 (0%)	1 (3.7%)		1 (20%)	0 (0%)	
		Ameloblastoma	0 (0%)	2 (7.4%)		0 (0%)	2 (8.3%)	
		Osteomyelitis	0 (0%)	2 (7.4%)		0 (0%)	2 (8.3%)	
		Keratocystic O. Tumor	0 (0%)	1 (3.7%)		0 (0%)	1 (4.2%)	
	Stage	T: (µ ± SD)	1.50 ± 0.71	3.15 ± 1.04	0.078	3.60 ± 0.89	2.82 ± 1.13	0.218
		N: (µ ± SD)	1 ± 1.41	0.45 ± 0.76	0.554	0.20 ± 0.45	0.59 ± 0.87	0.543
	Bone Defect	Length in cm: (µ ± SD)	6.50 ± 0.71	7.96 ± 1.97	0.156	9.20 ± 0.45	7.58 ± 2.01	**<0** **.** **001**
	Mucosa Defect	Length in cm: (µ ± SD)	5 ± 1.41	6.78 ± 2.54	0.344	8 ± 2.35	6.30 ± 2.47	0.089
		Width in cm: (µ ± SD)	3.50 ± 0.71	2.87 ± 1.10	0.373	3.40 ± 1.67	2.80 ± 0.89	0.137
	Overall Health	ASA: (µ ± SD)	2 ± 0	2.15 ± 0.77	0.768	2.40 ± 0.55	2.08 ± 0.78	0.482
Fibula Free Flap (FFF)-related Characteristics	Flap Type	Osteomyocutaneous	1 (50%)	21 (77.8%)	0.431	5 (100%)	17 (70.8%)	0.296
		Without Cutis	1 (50%)	6 (22.2%)		0 (0%)	7 (29.2%)	
	Reconstruction	Immediate	1 (50%)	20 (74.1%)	0.483	3 (60%)	18 (75%)	0.597
		Delayed	1 (50%)	7 (25.9%)		2 (40%)	6 (25%)	
	Fixation Method	MP	1 (50%)	23 (85.2%)	0.320	5 (100%)	19 (79.2%)	1.000
		RP	1 (50%)	2 (7.4%)		0 (0%)	3 (12.5%)	
		Wire	0 (0%)	2 (7.4%)		0 (0%)	2 (8.3%)	
	Fibula Segments	One Segment	2 (100%)	10 (37%)	0.325	2 (40%)	10 (41.7%)	0.695
		Two Segments	0 (0%)	10 (37%)		1 (20%)	9 (37.5%)	
		Three Segments	0 (0%)	7 (25.9%)		2 (40%)	5 (20.8%)	
		*N*: (µ ± SD)	1 ± 0	1.89 ± 0.80	0.177	2 ± 1	1.79 ± 0.78	0.304
	Fibula Osteotomies	Zero Osteotomies	1 (50%)	10 (37%)	1.000	1 (20%)	10 (41.7%)	0.356
		One Osteotomy	1 (50%)	12 (44.4%)		2 (40%)	11 (45.8%)	
		Two Osteotomies	0 (0%)	5 (18.5%)		2 (40%)	3 (12.3%)	
		*N*: (µ ± SD)	0.50 ± 0.71	0.81 ± 0.74	0.650	1.20 ± 0.84	0.71 ± 0.69	0.086
	Operative Time	Minutes: (µ ± SD)	513.50 ± 4.95	518.96 ± 115.60	0.474	515.20 ± 111.81	519.29 ± 113.73	0.471
	ICU Duration	Days: (µ ± SD)	1.50 ± 0.71	1.81 ± 1.86	0.897	3.80 ± 3.83	1.38 ± 0.58	0.115
Implant-related Characteristics	Implant System	BEGO®	0 (0%)	11 (40.7%)	0.594	1 (20%)	10 (41.7%)	0.497
		Xive®	2 (100%)	13 (48.1%)		3 (60%)	12 (50%)	
		Straumann®	0 (0%)	3 (11.1%)		1 (20%)	2 (8.3%)	
	Prosthetic Design	Fixed Bridge	1 (50%)	7 (25.9%)	0.483	1 (20%)	7 (29.2%)	1.000
		Telescopic Denture	1 (50%)	20 (74.1%)		4 (80%)	17 (70.8%)	
	Number of Implants	In Fibula: (µ ± SD)	3 ± 1.41	4.11 ± 2.03	0.228	4.60 ± 2.97	3.92 ± 1.79	0.248
		In Native Jaw: (µ ± SD)	2.50 ± 0.71	1.67 ± 1.94	0.315	2 ± 2.92	1.67 ± 1.69	0.363
		In Oppo. Jaw: (µ ± SD)	N/A	1.48 ± 2.85	0.650	1.40 ± 3.13	1.38 ± 2.76	1.000
		Sleeping Implants: (µ ± SD)	N/A	0.15 ± 0.36	0.768	0.20 ± 0.45	0.13 ± 0.34	0.801
		In Function: (µ ± SD)	2.50 ± 3.54	6.30 ± 3.99	0.102	7.40 ± 3.65	5.75 ± 4.11	0.207
		Failed Implants: (µ ± SD)	3 ± 4.24	0.41 ± 1.25	0.399	0.40 ± 0.89	0.63 ± 1.72	0.933
		Total Implants: (µ ± SD)	5.50 ± 0.71	7.26 ± 3.13	0.443	8 ± 2.92	6.96 ± 3.11	0.249
	Implant Survival	Failure	1 (50%)	4 (14.8%)	0.320	1 (20%)	4 (16.7%)	1.000
		Survival	1 (50%)	23 (85.2%)		4 (80%)	20 (83.3%)	

Mann–Whitney (U), independent samples *t*-test, and Fisher's exact test were used with a significance level of *p* < 0.05.

Bold values indicate statistically significant results.

At the donor site, higher age was significantly associated with WHD, with an approximately ten-year gap between those who had WHD and those who had no WHD (63.19 ± 8.4, 53.36 ± 13.34, *p* = 0.026), respectively. BMI, tobacco smoking, alcohol consumption, systemic comorbidities, tumor type and stage, bone and mucosal defects dimensions, and ASA score had no association with healing defects at donor site. Similarly, fibula type, reconstruction time, fixation method, number of segments, operative time and ICU durations, implant system, prosthetic design, and number of implants were not significantly associated with WHD at donor site (Table 6).

For PFL and revision surgery, age was associated with higher odds of incidence; however, the association was not statistically significant (*p* = 0.227 and 0.128, respectively). No other investigated variable was significantly linked to these two particular complications (Table 6).

### Speech, aesthetic, and nutrition

The majority of patients displayed intelligible speech (71.4%), whereas 25% were intelligible with concentration. Dental aesthetics was rated excellent in six patients (21.4%), good in twenty-one (75%) had good, and poor in one (3.6%). The mandibular-maxillary contour was rated satisfactory in 57.1% of cases, acceptable in 39.3% and unsatisfactory in 3.6%. One-quarter of the cohort (25%) reported a soft diet; the remaining patients reported a normal diet. There was no significant association between gender and speech, aesthetic or nutrition (Table 7).

**Table 7:** Post-operative outcomes of oncologic patients who underwent fibula free flap reconstruction in UKGM, January 2002–December 2010 (*n* = 29).

Variable	Outcome	Female (*n* = 9)	Male (*n* = 20)	Total (*n* = 29)	*p-value*
Speech	Unintelligible	1 (12.5%)	0 (0%)	1 (3.6%)	0.389
	Intelligible with concentration	2 (25.0%)	5 (25.0%)	7 (25.0%)	
	Intelligible	5 (62.5%)	15 (75.0%)	20 (71.4%)	
Dental Aesthetic	Poor	1 (12.5%)	0 (0%)	1 (3.6%)	0.383
	Good	6 (75.0%)	15 (75.0%)	21 (75.0%)	
	Excellent	1 (12.5%)	5 (25.0%)	6 (21.4%)	
Mandibular—Maxillary Contour	Unsatisfactory	1 (12.5%)	0 (0%)	1 (3.6%)	0.253
	Acceptable	2 (25.0%)	9 (45.0%)	11 (39.3%)	
	Satisfactory	5 (62.5%)	11 (55.0%)	16 (57.1%)	
Nutrition	Soft Diet	3 (37.5%)	4 (20.0%)	7 (25.0%)	0.371
	Normal Diet	5 (62.5%)	16 (80.0%)	21 (75.0%)	
Maximum inter-incisal opening	µ ± SD (Min-Max)	3.31 ± 1.36 (0.50–5.00)	4.38 ± 0.99 (2.50–6.00)	4.07 ± 1.18 (0.50–6.00)	**0** **.** **015**

Mann–Whitney (U) and Fisher's exact test were used with a significance level of *p* < 0.05.

Bold values indicate statistically significant results.

Delayed reconstruction, a higher number of failed implants, and lower implant survival were significantly associated with poorer speech performance (*p* = 0.047, 0.004 and 0.015, respectively). Similarly, the number of failed implants was significantly associated with lower dental-aesthetic scores (*p* = 0.032, Table 8).

**Table 8:** Risk factors of post-operative outcomes of oncologic patients who underwent fibula free flap reconstruction in UKGM, January 2002–December 2010 (*n* = 29).

Parameters	Variable	Outcome	Speech			*p-value*	Dental Aesthetic			*p- value*
			Unintelligible (*n* = 1)	Unintelligible w/ C. (*n* = 7)	Intelligible (*n* = 20)		Poor (*n* = 1)	Good (*n* = 21)	Excellent (*n* = 6)	
Demographic Characteristics	Age	Years: (µ ± SD)	62.75 ± 0	59.48 ± 7.25	55.76 ± 14.33	0.732	62.75 ± 0	59.56 ± 10.15	46.79 ± 17.14	0.079
		Months: (µ ± SD)	753 ± 0	713.71 ± 87.03	669.10 ± 171.98	0.732	753 ± 0	714.71 ± 121.76	561.50 ± 205.78	0.079
	Body Mass Index	BMI: (µ ± SD)	27.64 ± 0	27.78 ± 2.18	25.61 ± 4.12	0.402	27.64 ± 0	25.98 ± 3.45	26.84 ± 5.19	0.831
	Tobacco Smoking	Yes (*n*)	1 (100%)	3 (42.9%)	6 (30%)	0.468	1 (100%)	7 (33.3%)	2 (33.3%)	0.456
	Alcohol Drinking	Yes (*n*)	0 (0%)	1 (14.3%)	1 (5%)	0.497	0 (0%)	1 (4.8%)	1 (16.7%)	0.444
	Arterial Hypertension	Yes (*n*)	0 (0%)	3 (42.9%)	8 (40%)	1.000	0 (0%)	10 (47.6%)	1 (16.7%)	0.355
	CHD	Yes (*n*)	0 (0%)	1 (14.3%)	2 (10%)	1.000	0 (0%)	2 (9.5%)	1 (16.7%)	0.594
	COPD	Yes (*n*)	0 (0%)	1 (14.3%)	7 (35%)	0.738	0 (0%)	6 (28.6%)	2 (33.3%)	1.000
	Diabetes Mellitus	Yes (*n*)	0 (0%)	1 (14.3%)	1 (5%)	0.497	0 (0%)	2 (9.5%)	0 (0%)	1.000
	Thyroid G. Disorder	Yes (*n*)	0 (0%)	0 (0%)	2 (10%)	1.000	0 (0%)	2 (9.5%)	0 (0%)	1.000
Tumor-related Characteristics	Tumor Type	Squamous Cell Carcinoma	1 (100%)	6 (85.7%)	14 (70%)	0.751	1 (100%)	16 (76.2%)	4 (66.7%)	0.745
		Adenosquamous Carcinoma	0 (0%)	1 (14.3%)	0 (0%)		0 (0%)	1 (4.8%)	0 (0%)	
		Mucoepidermoid Carcinoma	0 (0%)	0 (0%)	1 (5%)		0 (0%)	1 (4.8%)	0 (0%)	
		Ameloblastoma	0 (0%)	0 (0%)	2 (10%)		0 (0%)	1 (4.8%)	1 (16.7%)	
		Osteomyelitis	0 (0%)	0 (0%)	2 (10%)		0 (0%)	1 (4.8%)	1 (16.7%)	
		Keratocystic O. Tumor	0 (0%)	0 (0%)	1 (5%)		0 (0%)	1 (4.8%)	0 (0%)	
	Stage	T: (µ ± SD)	1 ± 0	3.29 ± 0.95	3.08 ± 1.12	0.215	1 ± 0	3 ± 1.10	3.75 ± 0.50	0.119
		N: (µ ± SD)	N/A	0.71 ± 0.95	0.46 ± 0.78	0.651	N/A	0.50 ± 0.82	0.75 ± 0.96	0.713
	Bone Defect	Length in cm: (µ ± SD)	7 ± 0	8.71 ± 1.70	7.45 ± 1.90	0.293	7 ± 0	7.62 ± 1.82	8.33 ± 2.25	0.672
	Mucosa Defect	Length in cm: (µ ± SD)	4 ± 0	8 ± 2.58	6.24 ± 2.33	0.164	4 ± 0	6.68 ± 2.45	7 ± 2.92	0.562
		Width in cm: (µ ± SD)	3 ± 0	3.29 ± 0.76	2.77 ± 1.20	0.578	3 ± 0	3.11 ± 1.15	2.20 ± 0.45	0.212
	Overall Health	ASA: (µ ± SD)	2 ± 0	2.29 ± 0.76	2.15 ± 0.75	0.892	2 ± 0	2.19 ± 0.75	2.17 ± 0.75	0.941
Fibula Free Flap (FFF) Characteristics	Flap Type	Osteomyocutaneous	0 (0%)	7 (100%)	15 (75%)	0.080	0 (0%)	18 (85.7%)	4 (66.7%)	0.074
		Without Cutis	1 (100%)	0 (0%)	5 (25%)		1 (100%)	3 (14.3%)	2 (33.3%)	
	Reconstruction	Immediate	0 (0%)	7 (100%)	13 (65%)	**0** **.** **047**	0 (0%)	14 (66.7%)	6 (100%)	0.076
		Delayed	1 (100%)	0 (0%)	7 (35%)		1 (100%)	7 (33.3%)	0 (0%)	
	Fixation Method	MP	0 (0%)	7 (100%)	16 (80%)	0.237	0 (0%)	17 (81%)	6 (100%)	0.245
		RP	1 (100%)	0 (0%)	2 (10%)		1 (100%)	2 (9.5%)	0 (0%)	
		Wire	0 (0%)	0 (0%)	2 (10%)		0 (0%)	2 (9.5%)	0 (0%)	
	Fibula Segments	One Segment	1 (100%)	3 (42.9%)	8 (40%)	0.940	1 (100%)	10 (47.6%)	1 (16.7%)	0.371
		Two Segments	0 (0%)	3 (42.9%)	6 (30%)		0 (0%)	7 (33.3%)	2 (33.3%)	
		Three Segments	0 (0%)	1 (14.3%)	6 (30%)		0 (0%)	4 (19%)	3 (50%)	
		*N*: (µ ± SD)	1 ± 0	1.71 ± 0.76	1.90 ± 0.85	0.537	1 ± 0	1.71 ± 0.78	2.33 ± 0.82	0.158
	Fibula Osteotomies	Zero Osteotomies	0 (0%)	2 (28.6%)	9 (45%)	0.827	0 (0%)	9 (42.9%)	2 (33.3%)	0.750
		One Osteotomy	1 (100%)	3 (42.9%)	8 (40%)		1 (100%)	9 (42.9%)	2 (33.3%)	
		Two Osteotomies	0 (0%)	2 (28.6%)	3 (15%)		0 (0%)	3 (14.3%)	2 (33.3%)	
		*N*: (µ ± SD)	1 ± 0	1 ± 0.82	0.70 ± 0.73	0.641	1 ± 0	0.71 ± 0.72	1 ± 0.89	0.691
	Operative Time	Minutes: (µ ± SD)	517 ± 0	554.29 ± 121.05	511.75 ± 111.76	0.700	517 ± 0	497.57 ± 102.30	611 ± 114.27	0.084
	ICU Duration	Days: (µ ± SD)	1 ± 0	1.57 ± 0.79	1.95 ± 2.11	0.817	1 ± 0	2 ± 2.07	1.33 ± 0.52	0.615
FFF Complications	Partial Flap Loss	Yes	1 (100%)	0 (0%)	1 (5%)	0.071	1 (100%)	1 (4.8%)	0 (0%)	0.111
		No	0 (0%)	7 (100%)	19 (95%)		0 (0%)	20 (95.2%)	6 (100%)	
	WHD Recipient Site	Yes	1 (100%)	1 (14.3%)	7 (35%)	0.322	1 (100%)	5 (23.8%)	3 (50%)	0.195
		No	0 (0%)	6 (85.7%)	13 (65%)		0 (0%)	16 (76.2%)	3 (50%)	
	WHD Donor Site	Yes	0 (0%)	3 (42.9%)	6 (30%)	0.764	0 (0%)	8 (38.1%)	1 (16.7%)	0.747
		No	1 (100%)	4 (57.1%)	14 (70%)		1 (100%)	13 (61.9%)	5 (83.3%)	
	Revision Surgery	Yes	0 (0%)	2 (28.6%)	3 (15%)	0.655	0 (0%)	5 (23.8%)	0 (0%)	0.635
		No	1 (100%)	5 (71.4%)	17 (85%)		1 (100%)	16 (76.2%)	6 (100%)	
Implant-related Characteristics	Implant System	BEGO®	0 (0%)	5 (71.4%)	6 (30%)	0.100	0 (0%)	10 (47.6%)	1 (16.7%)	0.439
		Xive®	1 (100%)	1 (14.3%)	12 (60%)		1 (100%)	9 (42.9%)	4 (66.7%)	
		Straumann®	0 (0%)	1 (14.3%)	2 (10%)		0 (0%)	2 (9.5%)	1 (16.7%)	
	Prosthetic Design	Fixed Bridge	0 (0%)	1 (14.3%)	6 (30%)	0.725	0 (0%)	5 (23.8%)	2 (33.3%)	0.725
		Telescopic Denture	1 (100%)	6 (85.7%)	14 (70%)		1 (100%)	16 (76.2%)	4 (66.7%)	
	Number of Implants	In Fibula: (µ ± SD)	4 ± 0	5.57 ± 1.27	3.60 ± 2.01	0.073	4 ± 0	4.29 ± 2.17	3.50 ± 1.38	0.709
		In Native Jaw: (µ ± SD)	2 ± 0	1 ± 0.82	1.95 ± 2.19	0.542	2 ± 0	1.90 ± 2.10	1 ± 1.27	0.607
		In Oppo. Jaw: (µ ± SD)	N/A	1 ± 2.65	1.65 ± 2.96	0.775	N/A	0.95 ± 2.40	3.33 ± 3.72	0.165
		Sleeping Implants: (µ ± SD)	N/A	0.14 ± 0.38	0.15 ± 0.37	0.919	N/A	0.14 ± 0.36	0.17 ± 041	0.911
		In Function: (µ ± SD)	N/A	5.14 ± 4.78	6.75 ± 3.70	0.214	N/A	5.95 ± 3.78	7.67 ± 4.72	0.212
		Failed Implants: (µ ± SD)	6 ± 0	1.43 ± 2.23	0.05 ± 0.22	**0** **.** **004**	6 ± 0	0.52 ± 1.40	N/A	**0** **.** **032**
		Total Implants: (µ ± SD)	6 ± 0	7.57 ± 2.57	7.20 ± 3.32	0.890	6 ± 0	7.14 ± 2.58	7.83 ± 4.79	0.825
	Implant Survival	Failure	1 (100%)	3 (42.9%)	1 (5%)	**0** **.** **015**	0 (0%)	17 (81%)	6 (100%)	0.143
		Survival	0 (0%)	4 (57.1%)	19 (95%)		1 (100%)	4 (19%)	0 (0%)	

Parameters	Variable	Outcome	Mandibular—Maxillary Contour			*p-value*	Nutrition		*p-value*
			Unsatisfactory (*n* = 1)	Acceptable (*n* = 11)	Satisfactory (*n* = 16)		Soft Diet (*n* = 7)	Normal Diet (*n* = 21)	
Demographic Characteristics	Age	Years: (µ ± SD)	62.75 ± 0	57.51 ± 11.58	56.18 ± 14	0.079	59.30 ± 7.71	56.15 ± 13.99	0.289
		Months: (µ ± SD)	753 ± 0	690.09 ± 138.92	674.19 ± 167.95	0.079	711.57 ± 92.56	673.81 ± 167.92	0.289
	Body Mass Index	BMI: (µ ± SD)	27.64 ± 0	26.58 ± 3.06	25.89 ± 4.31	0.831	27.80 ± 2.71	25.70 ± 3.94	0.102
	Tobacco Smoking	Yes (*n*)	1 (100%)	2 (18.2%)	7 (43.8%)	0.108	3 (42.9%)	7 (33.3%)	0.674
	Alcohol Drinking	Yes (*n*)	0 (0%)	1 (9.1%)	1 (6.3%)	1.000	1 (14.3%)	1 (4.8%)	0.444
	Arterial Hypertension	Yes (*n*)	0 (0%)	5 (45.5%)	6 (37.5%)	0.824	2 (28.6%)	9 (42.9%)	0.668
	Coronary Heart Disease	Yes (*n*)	0 (0%)	2 (18.2%)	1 (6.3%)	0.597	1 (14.3%)	2 (9.5%)	1.000
	COPD	Yes (*n*)	0 (0%)	3 (27.3%)	5 (31.3%)	1.000	2 (28.6%)	6 (28.6%)	1.000
	Diabetes Mellitus	Yes (*n*)	0 (0%)	2 (18.2%)	0 (0%)	0.217	1 (14.3%)	1 (4.8%)	0.444
	Thyroid Gland Disorder	Yes (*n*)	0 (0%)	0 (0%)	2 (12.5%)	0.534	0 (0%)	2 (9.5%)	1.000
Tumor-related Characteristics	Tumor Type	Squamous Cell Carcinoma	1 (100%)	9 (81.8%)	11 (68.8%)	0.832	6 (85.7%)	15 (71.4%)	0.650
		Adenosquamous Carcinoma	0 (0%)	1 (9.1%)	0 (0%)		1 (14.3%)	0 (0%)	
		Mucoepidermoid Carcinoma	0 (0%)	0 (0%)	1 (6.3%)		0 (0%)	1 (4.8%)	
		Ameloblastoma	0 (0%)	1 (9.1%)	1 (6.3%)		0 (0%)	2 (9.5%)	
		Osteomyelitis	0 (0%)	0 (0%)	2 (12.5%)		0 (0%)	2 (9.5%)	
		Keratocystic Odontogenic Tumor	0 (0%)	0 (0%)	1 (6.3%)		0 (0%)	1 (4.8%)	
	Stage	T: (µ ± SD)	1 ± 0	3.13 ± 0.99	3.17 ± 1.12	0.076	2.57 ± 1.13	3.29 ± 1.07	0.087
		N: (µ ± SD)	N/A	0.88 ± 0.99	0.33 ± 0.65	0.713	1 ± 1	0.29 ± 0.61	0.149
	Bone Defect	Length in cm: (µ ± SD)	7 ± 0	7.55 ± 1.13	7.94 ± 2.32	0.672	7.43 ± 0.98	7.86 ± 2.10	0.305
	Mucosa Defect	Length in cm: (µ ± SD)	4 ± 0	6.73 ± 2.37	6.77 ± 2.68	0.562	5.71 ± 2.56	7 ± 2.45	0.128
		Width in cm: (µ ± SD)	3 ± 0	3.09 ± 0.94	2.77 ± 1.24	0.502	3.14 ± 0.90	2.83 ± 1.15	0.265
	Overall Health	ASA: (µ ± SD)	2 ± 0	2.27 ± 0.65	2.13 ± 0.81	0.969	2.29 ± 0.76	2.14 ± 0.73	0.330
Fibula Free Flap (FFF)-related Characteristics	Flap Type	Osteomyocutaneous	0 (0%)	10 (90.9%)	12 (75%)	0.175	6 (85.7%)	16 (76.2%)	1.000
		Without Cutis	1 (100%)	1 (9.1%)	4 (25%)		1 (14.3%)	5 (23.8%)	
	Reconstruction	Immediate	0 (%)	8 (72.7%)	12 (75%)	0.433	6 (85.7%)	14 (66.7%)	0.404
		Delayed	1 (100%)	3 (27.3%)	4 (25%)		1 (14.3%)	7 (33.3%)	
	Fixation Method	MP	0 (0%)	10 (90.9%)	13 (81.3%)	0.156	6 (85.7%)	17 (81%)	1.000
		RP	1 (100%)	0 (0%)	2 (12.5%)		1 (14.3%)	2 (9.5%)	
		Wire	0 (0%)	1 (9.1%)	1 (6.3%)		0 (0%)	2 (9.5%)	
	Fibula Segments	One Segment	1 (100%)	6 (54.5%)	5 (31.3%)	**0** **.** **041**	4 (57.1%)	8 (38.1%)	0.248
		Two Segments	0 (0%)	5 (45.5%)	4 (25%)		3 (42.9%)	6 (28.6%)	
		Three Segments	0 (0%)	0 (0%)	7 (43.8%)		0 (0%)	7 (33.3%)	
		*N*: (µ ± SD)	1 ± 0	1.45 ± 0.52	2.12 ± 0.89	0.081	1.43 ± 0.54	1.95 ± 0.87	0.208
	Fibula Osteotomies	Zero Osteotomies	0 (0%)	4 (36.4%)	7 (43.8%)	0.589	3 (42.9%)	8 (38.1%)	0.446
		One Osteotomy	1 (100%)	6 (54.5%)	5 (31.3%)		4 (57.1%)	8 (38.1%)	
		Two Osteotomies	0 (0%)	1 (9.1%)	4 (25%)		0 (0%)	5 (23.8%)	
		*N*: (µ ± SD)	1 ± 0	0.73 ± 0.65	0.81 ± 0.83	0.910	0.57 ± 0.54	0.86 ± 0.79	0.499
	Operative Time	Minutes: (µ ± SD)	517 ± 0	490.91 ± 93.65	544.69 ± 123.30	0.084	515.86 ± 61.59	524.81 ± 124.79	0.429
	ICU Duration	Days: (µ ± SD)	1 ± 0	1.45 ± 0.52	2.12 ± 2.36	0.735	1.29 ± 0.49	2 ± 2.07	0.499
FFF Complications	Partial Flap Loss	Yes	1 (100%)	1 (9.1%)	0 (0%)	**0** **.** **029**	2 (28.6%)	0 (0%)	0.056
		No	0 (0%)	10 (90.9%)	16 (100%)		5 (71.4%)	21 (100%)	
	WHD Recipient Site	Yes	1 (100%)	1 (9.1%)	7 (43.8%)	**0** **.** **037**	1 (14.3%)	8 (38.1%)	0.371
		No	0 (0%)	10 (90.9%)	9 (56.3%)		6 (85.7%)	13 (61.9%)	
	WHD Donor Site	Yes	0 (0%)	3 (27.3%)	6 (37.5%)	0.791	1 (14.3%)	8 (38.1%)	0.371
		No	1 (100%)	8 (72.7%)	10 (62.5%)		6 (85.7%)	13 (61.9%)	
	Revision Surgery	Yes	0 (0%)	2 (18.2%)	3 (18.8%)	1.000	0 (0%)	5 (23.8%)	0.290
		No	1 (100%)	9 (81.8%)	13 (81.3%)		7 (100%)	16 (76.2%)	
Implant Characteristics	Implant System	BEGO®	0 (0%)	6 (54.5%)	5 (31.3%)	0.715	4 (57.1%)	7 (33.3%)	0.569
		Xive®	1 (100%)	4 (36.4%)	9 (56.3%)		3 (42.9%)	11 (52.4%)	
		Straumann®	0 (0%)	1 (9.1%)	2 (12.5%)		0 (0%)	3 (14.3%)	
	Prosthetic Design	Fixed Bridge	0 (0%)	3 (27.3%)	4 (25%)	1.000	1 (14.3%)	6 (28.6%)	0.639
		Telescopic Denture	1 (100%)	8 (72.7%)	12 (75%)		6 (85.7%)	15 (71.4%)	
	Number of Implants	In Fibula: (µ ± SD)	4 ± 0	4.18 ± 1.78	4.06 ± 2.24	0.709	3.71 ± 2.06	4.24 ± 2	0.278
		In Native Jaw: (µ ± SD)	2 ± 0	1.91 ± 1.81	1.56 ± 2.10	0.607	2.43 ± 1.72	1.48 ± 1.97	0.113
		In Oppo. Jaw: (µ ± SD)	N/A	1.27 ± 2.83	1.63 ± 2.94	0.841	1 ± 2.65	1.57 ± 2.91	0.756
		Sleeping Implants: (µ ± SD)	N/A	0.09 ± 0.30	0.19 ± 0.40	0.917	N/A	0.19 ± 0.40	0.466
		In Function: (µ ± SD)	N/A	5.36 ± 4.74	7 ± 3.35	0.212	4.29 ± 4.92	6.71 ± 3.69	0.088
		Failed Implants: (µ ± SD)	6 ± 0	0.91 ± 1.87	0.06 ± 0.25	**0** **.** **019**	2 ± 2.83	0.14 ± 0.48	0.155
		Total Implants: (µ ± SD)	6 ± 0	7.36 ± 2.98	7.25 ± 3.28	0.825	7.14 ± 2.73	7.29 ± 3.21	0.458
	Implant Survival	Failure	0 (0%)	3 (27.3%)	1 (6.3%)	0.053	3 (42.9%)	2 (9.5%)	0.082
		Survival	1 (100%)	8 (72.7%)	15 (93.8%)		4 (57.1%)	19 (90.5%)	

Kruskal–Wallis (H), Mann–Whitney (U), one-way analysis of variance (ANOVA), independent samples *t*-test, and Fisher's exact test were used with a significance level of *p* < 0.05.

Bold values indicate statistically significant results.

A higher number of fibula segments and fewer failed implants were significantly associated with improved perceptions of mandibular-maxillary contour (*p* = 0.041 and 0.019). PFL and recipient-site WDH were significantly associated with lower levels of mandibular-maxillary contour (*p* = 0.029 and 0.037). Patients adhering to a soft diet tended to be older (59.30 vs. 56.15), to have a higher BMI (27.80 vs. 25.70 kg/m^2^), to experience a higher incidence of PFL (28.6% vs. 0%), and to show a higher mean number of failed implants (2 vs. 0.14), without statistical significance (Table 8).

### Maximum inter-incisal opening (MIO)

The average MIO was 4.07 ± 1.18 cm (0.5–6). Male patients showed significantly greater MIO than female patients (4.38 ± 0.99 cm and 3.31 ± 1.36 cm, *p* = 0.015, Table 7).

**Figure 2:**
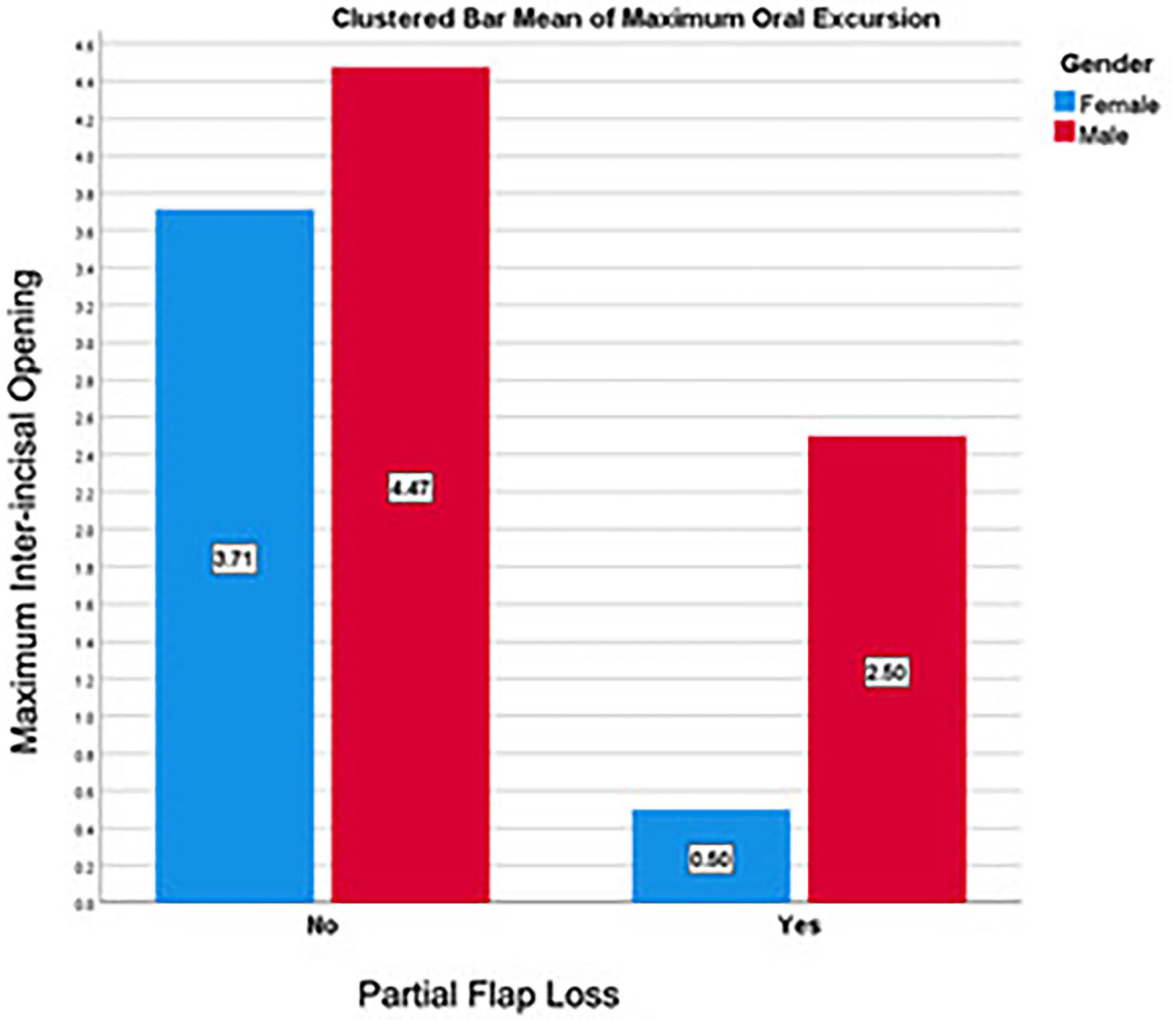
Maximum inter-incisal opening Among oncologic patients who underwent fibula free flap reconstruction in UKGM, stratified by gender and PFL, January 2002–December 2010 (*n* = 29).

Patients with PFL exhibited a significantly lower MIO than those without PFL (1.50 ± 1.41 cm, 4.27 ± 0.93 cm, *p* < 0.001, Figure 2). Recipient-site WHD were likewise associated with a reduced MIO (3.50 cm vs. 4.34 cm, *p* = 0.039). Patients whose speech was intelligible demonstrated the highest MIO, compared to those intelligible with concentration and those with unintelligible speech (4.25 ± 0.98 cm, 4.07 ± 1.02 cm and 0.5 ± 0 cm, *p* = 0.004). Similarly, patients who perceived excellent dental aesthetic demonstrated the highest MIO compared with good and poor aesthetics (4.42 ± 1.20 cm, 4.14 ± 0.92 cm and 0.5 ± 0 cm, respectively, *p* = 0.004, Table 9).

**Table 9:** Predictors of Maximum inter-incisal opening (MIO) of oncologic patients who underwent fibula free flap reconstruction in UKGM, January 2002–December 2010 (*n* = 29).

Variable	Outcome	MIO (4.07 ± 1.18)	*p-value*
Tobacco Smoking	Yes	4.35 ± 1.49	0.182
	No	3.92 ± 0.99	
Alcohol Drinking	Yes	4 ± 0	0.894
	No	4.08 ± 1.23	
Arterial Hypertension	Yes	4.18 ± 1.03	0.350
	No	4 ± 1.30	
Coronary Heart Disease	Yes	4.67 ± 0.58	0.183
	No	4 ± 1.22	
COPD	Yes	4.19 ± 1.10	0.375
	No	4.03 ± 1.24	
Diabetes Mellitus	Yes	4.25 ± 1.06	1.000
	No	4.06 ± 1.21	
Thyroid Gland Disorder	Yes	4 ± 0	0.894
	No	4.08 ± 1.23	
Tumor Type	Squamous Cell Carcinoma	3.93 ± 1.26	0.695
	Adenosquamous Carcinoma	4 ± 0	
	Mucoepidermoid Carcinoma	4 ± 0	
	Ameloblastoma	4.25 ± 1.06	
	Osteomyelitis	4.50 ± 0.71	
	Keratocystic Odontogenic Tumor	6 ± 0	
Flap Type	Osteomyocutaneous	4.14 ± 0.98	0.294
	Without Cutis	3.83 ± 1.86	
Reconstruction	Immediate	4 ± 1.63	0.422
	Delayed	4.10 ± 1.01	
Fixation Method	MP	4.30 ± 1.02	0.055
	RP	2.67 ± 1.89	
	Wire	3.50 ± 0	
Fibula Segments	One Segment	3.50 ± 1.37	0.054
	Two Segments	4.72 ± 0.75	
	Three Segments	4.21 ± 0.91	
Fibula Osteotomies	Zero Osteotomies	3.86 ± 1.19	0.322
	One Osteotomy	3.95 ± 1.34	
	Two Osteotomies	4.80 ± 0.45	
Implant System	BEGO®	3.73 ± 0.93	0.112
	Xive®	4.07 ± 1.31	
	Straumann®	5.33 ± 0.58	
Prosthetic Design	Fixed Bridge	3.93 ± 1.27	0.360
	Telescopic Denture	4.12 ± 1.18	
Implant Survival	Failure	3.30 ± 1.72	0.055
	Survival	4.24 ± 1.01	
Partial Flap Loss	Yes	1.50 ± 1.41	**<0.001**
	No	4.27 ± 0.93	
Wound Healing Defect: Recipient Site	Yes	3.50 ± 1.52	**0.039**
	No	4.34 ± 0.91	
Wound Healing Defect: Donor Site	Yes	4.06 ± 0.85	0.481
	No	4.08 ± 1.34	
Revision Surgery	Yes	4.30 ± 1.10	0.321
	No	4.02 ± 1.22	
Speech	Unintelligible	0.50 ± 0	**0.004**
	Intelligible with concentration	4.07 ± 1.02	
	Intelligible	4.25 ± 0.98	
Dental Aesthetic	Poor	0.50 ± 0	**0.004**
	Good	4.14 ± 0.92	
	Excellent	4.42 ± 1.20	
Mandibular—Maxillary Contour	Unsatisfactory	0.50 ± 0	**0.001**
	Acceptable	3.82 ± 0.93	
	Satisfactory	4.47 ± 0.94	
Nutrition	Soft Diet	3.21 ± 1.44	**0.012**
	Normal Diet	4.36 ± 0.96	

Mann–Whitney (U), one-way analysis of variance (ANOVA), and independent samples *t*-test were used with a significance level of *p* < 0.05.

Bold values indicate statistically significant results.

**Figure 3:**
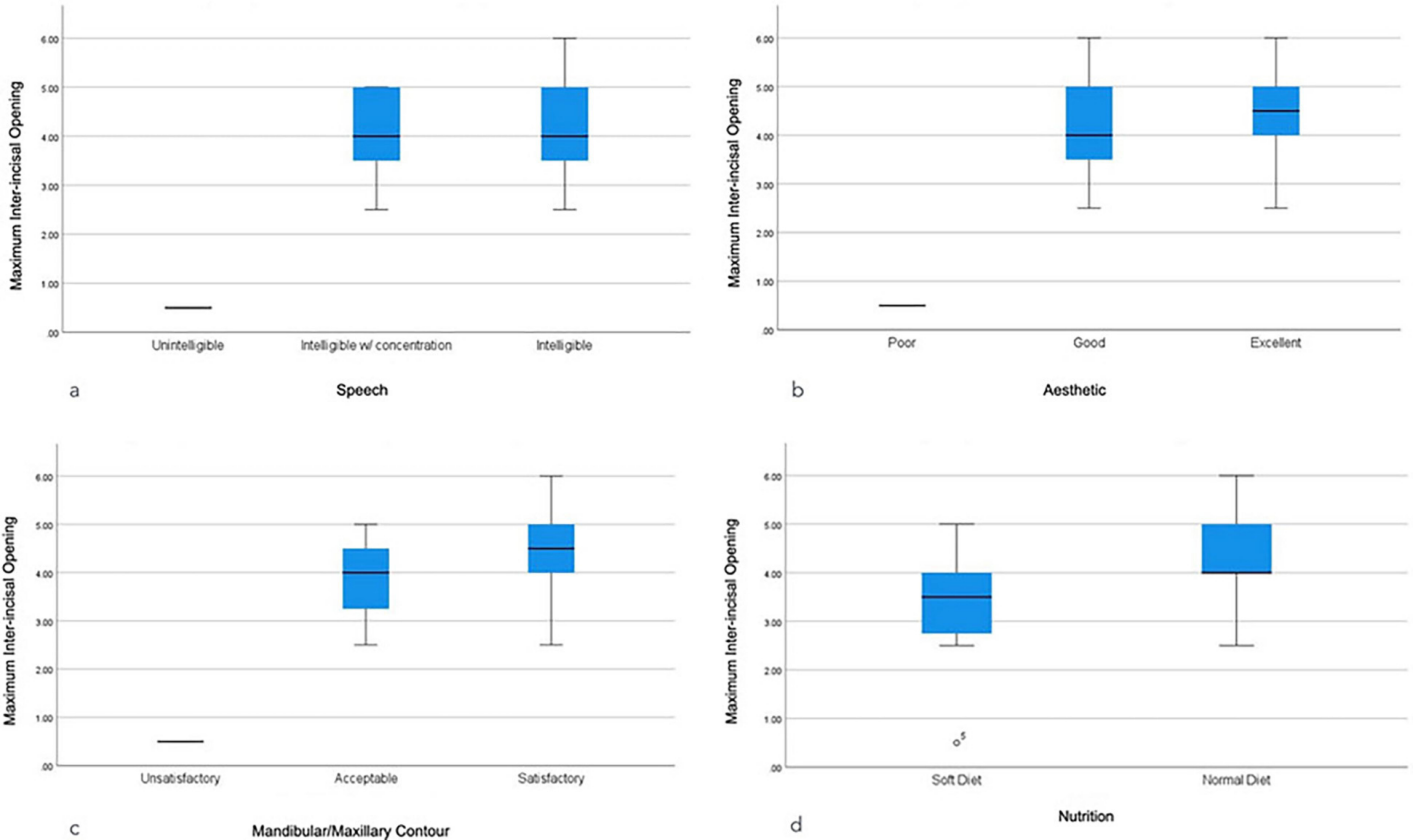
Maximum inter-incisal opening Among oncologic patients who underwent fibula free flap reconstruction in UKGM, stratified by **(a)** speech, **(b)** dental aesthetic, **(c)** mandibular—maxillary contour, and **(d)** nutrition, January 2002–December 2010 (*n* = 29).

A satisfactory mandibular-maxillary contour yielded the highest MIO, exceeding acceptable and unsatisfactory contours (4.47 ± 0.94 cm, 3.82 ± 0.93 and 0.5 ± 0, respectively, *p* = 0.001). Finally, consuming a soft diet was significantly associated with a lower MIO compared with a normal diet (3.21 ± 1.44 cm and 4.36 ± 0.96 cm, respectively, *p* = 0.012, Figure 3).

### Mandibular-Maxillary contour

Our analysis yielded pseudo R-squared values of 21.6% (McFadden's R2) and 26.3% (Nagelkerke's R2), indicating that these variables had moderate predictive power. Notably, our results showed that operative time did not significantly predict contour level, whereas the number of fibula segments was a significant predictor (*p* = 0.806 and <0.001, respectively). Specifically, our model estimated that having one fibula segment was associated with an adjusted decrease in contour level of 12.99 compared to having three segments, while having two segments was associated with an adjusted decrease of 12.74 compared to having three segments (Table 10).

**Table 10:** Ordinal logistic regression for mandibular-maxillary contour Among oncologic patients who underwent fibula free flap reconstruction in UKGM, January 2002–December 2010 (*n* = 29).

Predictor	Estimate	SE	Z	*p-value*
Operative Time	−0.00132	0.00537	−0.245	0.806
Fibula Segments: 1 vs. 3	−12.99401	1.08273	−12.001	**<0.001**
Fibula Segments: 2 vs. 3	−12.73819	1.01178	−12.590	**<0.001**

McFadden's R^2^ (R^2^_McF_) = 0.216; Nagelkerke's R^2^ (R^2^_N_) = 0.263; AIC = 45.4; Deviance = 35.4.

Bold values indicate statistically significant results.

## Discussion

The present study provides an integrated view of flap morbidity, implant performance and patient-centered function after mandibular reconstruction with a vascularized fibula free flap.

### Flap-related morbidity

A recipient-site WHD rate of 31% accords with the 33%–35% reported by Rendenbach et al. in a 128-patient series of osseous free flaps (19). Multivariable analysis confirmed that multisegment osteotomies and extended operative time were independent predictors of WHD in our cohort, which aligns with prior reports (19, 20). Revision surgery was significantly more frequent when bone defects were larger, implicating defect length as an additional driver of morbidity (20). These observations underscore that every additional osteotomy introduces vascular and mechanical vulnerability and should therefore be balanced against the aesthetic advantage of improved contour. In surgical planning, the segment count should be therefore considered as modifiable risk factor.

Donor-site WHD occurred in 31% of patients and increased with age, whereas a recent multicenter study linked donor-site morbidity chiefly to alcohol abuse and skin-graft closure, without an age effect (13). Divergent risk profiles may reflect institutional differences in flap procurement and protocols.

Total flap failure rates of 3.1%–14.4% have been documented, yet partial failures appear to be underreported in the literature (21, 22). Even limited tissue loss may foster infections and delay adjuvant therapy or prosthetic rehabilitation (21). In the present cohort, PFL was infrequent (6.9%), but had clear downstream consequences, being associated with both reduced MIO and poorer perceived contour. Restriction to patients who ultimately received dental implant rehabilitation introduces a flap-specific selection bias that may have excluded early losses and thereby understated true incidence, without affecting implant and functional analyses.

### Clinical and functional outcomes of implant rehabilitation

The insertion of dental implants following jaw reconstruction with FFF was shown to be a successful option for oral rehabilitation in patients with oral cancer (5). Favorable implant survival rates of implants placed in FFF align with recent meta-analytic reports and even contemporary “jaw-in-a-day” protocols that report implant survivals of ≥95% (23, 24). In our cohort, each patient received an average of 7.14 ± 3.06 implants, and fewer than one implant per patient failed on average. Most patients were rehabilitated with telescopic prostheses, a modality that allows non-integrated elements to be excluded without sacrificing the superstructure. This design choice may partly explain why patients with one or more failed implants still reported acceptable functional outcomes and aesthetics.

Over 71% of participants demonstrated intelligible speech, 75% rated their dental aesthetics as good and over 21% rated them excellent, echoing the good-to-excellent speech intelligibility and aesthetics after reconstruction of segmental maxillofacial defects with FFF (25). Failed implants mattered clinically, and a higher number of failed implants and lower implant survival were associated with reduced speech intelligibility and poorer aesthetic rankings (6). Consistent with this association, prosthetic protocols for higher-risk patients should prioritize flexibility. Removable designs, such as telescopic overdentures, can be advantageous and can be leveraged to maintain function if an implant is lost.

Regarding MIO, the present study demonstrates that WHDs and PFL each diminish MIO by approximately one and three centimeters, respectively, an effect size likely to translate into dietary limitation, given the concurrent association between lower MIO and soft-diet dependence in included sample. Importantly, MIO fell stepwise with declining speech intelligibility, dental aesthetic ratings and contour scores, reinforcing its utility as an objective surrogate of oral function. Accordingly, rehabilitation should include early trismus prophylaxis and structured physiotherapy, and prosthetic selection should accommodate limited mouth opening in patients with WHD or PFL.

Three-segment osteotomies increased WHD risk. At the same time, they were the only independent predictor of a superior contour score. Contemporary computer-aided planning studies advocate multisegment designs to restore native angles and jaw shape; our findings caution that this aesthetic gain must be balanced against higher soft-tissue morbidity (26). The risk-benefit trade-off aligns with a recent comprehensive review that highlights multifactorial determinants of implant survival and the cumulative impact of co-occuring risk factors, underscoring the need for comprehensive risk assessment and personalized treatment planning (10).

Hölzle et al. reported poor recipient-site aesthetics in 62% of women vs. 34% of men after fibula reconstruction, despite greater functional satisfaction in women (27). Their finding is tempered by the fact that only 43% of that cohort received implant-supported restorations, a factor known to improve facial contours and oral function (28, 29). Implant rehabilitation after FFF reconstruction thus remains infrequent (1, 27, 30). By contrast, every patient in the present study received implants, allowing direct linkage of demographic, oncologic and flap variables, complications and implant metrics with both objective functional and patient-reported outcomes. Here, women exhibited more failed implants (1.67 vs. 0.10) without statistical significance, and no sex-related differences emerged in speech intelligibility, aesthetics, contour or diet. The lower MIO in female patients aligns with published sex differences in maximal mouth opening in non-reconstructed populations (31).

### Limitations

The study's retrospective design, modest sample size and single-center setting constrain generalizability. Moreover, covariates such as radiation dose and fields, timing, osteotomy gap width or defect geometry, environmental factors, and comorbidities may not have been fully captured or may have influenced outcomes independently of flap-related factors. Complex defects were more likely to receive multisegment osteotomies and longer operative times, introducing potential confounding by indication. Furthermore, restricting the cohort to patients who underwent implant rehabilitation may underestimate early flap-related failures.

### Future directions

Well-powered, prospective, multicenter registries that record granular variables such as radiotherapy dose, segment vascular pattern and closure technique are required to refine risk calculators for WHD and implant failure. Although an encouraging 38-year case report of fibula reconstruction documents a patient satisfied with functional outcomes, normal diet, speech and head movement (32), systematic analyses emphasize that high-quality, long-term, standardized comparative studies are still needed (33). Prospective cohorts with extended follow-up are therefore indispensable to confirm the durability of functional and aesthetic outcomes in the long term.

## Conclusion

Implant-supported restorations provide a dependable avenue for oral rehabilitation following FFF jaw reconstruction. Recipient-site WHD and PFL were more frequent with multisegment osteotomies and extended operative time, and they reduced MIO and contour scores. Implant failure lowered speech intelligibility and aesthetic ratings. Nevertheless, 96% of patients spoke intelligibly, with or without concentration, 96% rated their dental aesthetic as good or excellent, and most ate a normal diet. These results address the study aim by clarifying how flap morbidity translates into clinical and functional implant outcomes. Long-term success therefore depends on balancing the contour benefits of complex osteotomies with the biological tolerance of the flap after jaw reconstruction.

## Data Availability

The raw data supporting the conclusions of this article will be made available by the authors, without undue reservation.
